# The ACE genes in *Aphelenchoides besseyi* isolates and their expression correlation to the fenamiphos treatment

**DOI:** 10.1038/s41598-022-05998-y

**Published:** 2022-02-07

**Authors:** Jung-Kai Hsu, Chia-Wei Weng, Jeremy J. W. Chen, Peichen Janet Chen

**Affiliations:** 1 Department of Plant Pathology, National Chung Hsing University, Taichung, Taiwan; 2 Institute of Medicine, Chung Shan Medical University, Taichung, Taiwan; 3 Institute of Biomedical Sciences, National Chung Hsing University, Taichung, Taiwan

**Keywords:** Enzyme mechanisms, Mechanism of action

## Abstract

*Aphelenchoides besseyi* could cause great yield losses of rice and many economically important crops. Acetylcholinesterase (AChE) inhibitors were commonly used to manage plant-parasitic nematodes. However, nematodes resistant to AChE inhibitors have been increasingly reported due to the extensive use of these chemicals. The current study was aimed to establish the correlation between fenamiphos (an AChE-inhibitor) sensitivities and acetylcholinesterase genes (*ace*) by analyzing two isolates of *A. besseyi* (designated Rl and HSF), which displayed differential sensitivities to fenamiphos. The concentrations of fenamiphos that led to the death of 50% (LD_50_) of Rl and HSF were 572.2 ppm and 129.4 ppm, respectively. Three *ace* genes were cloned from *A. besseyi* and sequenced. Sequence searching and phylogenic analyses revealed that AChEs of R1 and HSF shared strong similarities with those of various vertebrate and invertebrate species. Molecular docking analysis indicated that AChEs-HSF had much higher affinities to fenamiphos than AChEs-R1. Quantitative reverse transcriptase-PCR analyses revealed that expression of three *ace* genes were downregulated in HSF but were upregulated in Rl after exposure to 100 ppm fenamiphos for 12 h. The results indicated that the expression of the *ace* genes was modulated in response to fenamiphos in different nematode strains. An increased expression of the *ace* genes might contribute to fenamiphos-insensitivity as seen in the Rl isolate.

## Introduction

Plant parasitic nematodes have been estimated to cause $US16 billion-dollar worth of yield losses in rice annually worldwide^1^. The rice white tip nematode, *Aphelenchoides besseyi*, accounts for 10–30% of the total damage to rice crops. The nematode has been reported to cause greater than 70% yield losses on highly susceptible rice varieties^2,3^. In addition to rice, *A. besseyi* can be found in many economically important crops including strawberry, chrysanthemum, and bird’s-nest fern. It is difficult to control *A. besseyi* as it has a wide host range and can feed on fungi^4–6^.

Soaking rice seeds with nematicides is the most common strategy to control *A. besseyi*. Nematicides including organophosphates (OPs) and carbamates (CBs) that inhibit the acetylcholinesterase (AChE, EC 3.1.1.7) activity are often recommended to control *A. besseyi*. AChE-inhibiting nematicides are widely used in agricultural pest managements because of their high efficacy and low cost^7^. AChE-inhibitors would compete with acetylcholine, inhibit cholinesterase to hydrolyze acetylcholine, and eventually leads to the accumulation of excess acetylcholine, which disrupts the neurotransmission pathway, thereby preventing nematodes from finding and up-taking food, causing overly convulsion and leading to mortality in nematodes^8,9^. However, a low nematode mortality rate has been observed in rice seeds even after being soaked with this nematicide, likely due to the protection of rice glumes or the occurrence of nematicide-resistant nematodes^10^. Pesticide resistance has been a concerning issue since mid-1940s because of the extensive and repeated use of chemical agents^11,12^. Although the mode of action of AChE-inhibiting insecticides has been intensively studied in insects, little is known about the mechanisms by which plant-parasitic nematodes confer resistance to the AChE inhibitors.

Target site insensitivity is one of the most common mechanisms leading to pesticide resistance, which could cause the difficulty in pest management^12–15^. Point mutations in various positions of AChE of insects have been reported to result in resistance to AChE inhibiting insecitides^15^. Organophosphate and carbamate insecticides are commonly used to control *Anopheles* spp., the major mosquito vectors of malaria. Mosquitoes resistant to organophosphate and carbamate have been found to be resulted from a point mutation in AChE1 (G119S), which leads to a reduced susceptibility to AChE-inhibitors^16–18^. Resistance to benzimidazoles in the animal parasitic nematode *Haemonchus contortus* is the result of point mutations (F200Y with F167Y or E198A) within β-tubulin^19^. The presence of 18 point mutations in AChE2 has also been shown to be associated with the fosthiazate resistance in the plant-parasitic nematode, *M. incognita*^20^.

In addition to target site insensitivity, over-accumulation of neuronal and non-neuronal AChEs could confer resistance to AChE-inhibitors^21^. Overexpression of neuronal AChE is resulted from transcriptional activation and duplication of the *ace* genes. As the amount of AChE increases, the pests could become more resistant to AChE-inhibitors^22^. For instance, increased expression of a single copy of the *ace* gene in *Drosophila* sp. has been shown to lead to parathion resistance^23^. Similar phenomena have been found in organophosphate-resistant greenbug (*Schizaphis graminum*)^24^. Duplication of the *ace* genes has been found in some cases of insecticide-resistance, which often results in higher AChE catalytic efficiency and resistance to AChE-inhibitors^21,25,26^. Non-neuronal AChE affects the function of AChE-inhibitors, thereby reducing cellular sensitivity to AChE-inhibitors^21^. Mammalian cells become more resistant to organophosphate as the result of the non-neuronal read-through AChE (termed AChE-R), which could repair neurodegeneration and avoid cell damage upon exposure to organophosphate^27^. Studies have also found that both *Caenorhabditis elegans* and *Bursaphelenchus xylophilus* nematodes have a non-neuronal AChE3 gene, which has been shown to be responsible for resistance to xenobiotic substances^28–30^. How plant-parasitic nematodes are resistant to the AChE inhibitors remains largely unknown.

Two *A. besseyi* isolates designated R1 and HSF were isolated from rice and bird-nest fern, respectively. Sensitivity tests revealed a drastic difference between these two isolates, in which the R1 isolate was highly tolerant and the HSF isolate was sensitive to fenamiphos and carbofuran, both of which are AChE-inhibiting nematicides. In the present study, the *A. besseyi* acetylcholinesterase-coding genes (*Abace*) of two isolates were identified and sequenced. The 3D-structures of AChEs were revealed based on computational predictions. Molecular docking analysis of AChEs was performed to predict mutations in AChE and to establish a possible link with fenamiphos resistance. Finally, qRT-PCR was applied to determine the expression levels of the *ace* genes in R1 and HSF isolates before and after fenamiphos treatment. Taken together, our results indicated that mutations in the acetylcholinesterase-coding genes leading to different affinities of AChEs to fenamiphos and differential expression of the *Abace* genes may be associated with resistance/susceptibility to fenamiphos in *A. besseyi*.

## Results

### Identification of nematode species

Both Rl and HSF isolates used in this study were identified as *Aphelenchoides besseyi* based on 18S rRNA gene sequence similarity and morphological characteristics. Both nematode isolates have morphological characteristics resembling *Aphelenchoides* spp., all having offset lip region. The metacorpus width was found to be larger than 75% body width, pharyngeal glands overlapping ventrally and 3–4 mucros at the tail tip^31^. The 18S rRNA gene sequences of the Rl and HSF isolates had 100% and 99.56% identities (with zero E value) to those of *Aphelenchoides besseyi* (#KT454963 and #KT454962), respectively.

### *A. besseyi* isolates display different sensitivities to the AChE inhibitor

**Figure 1:**
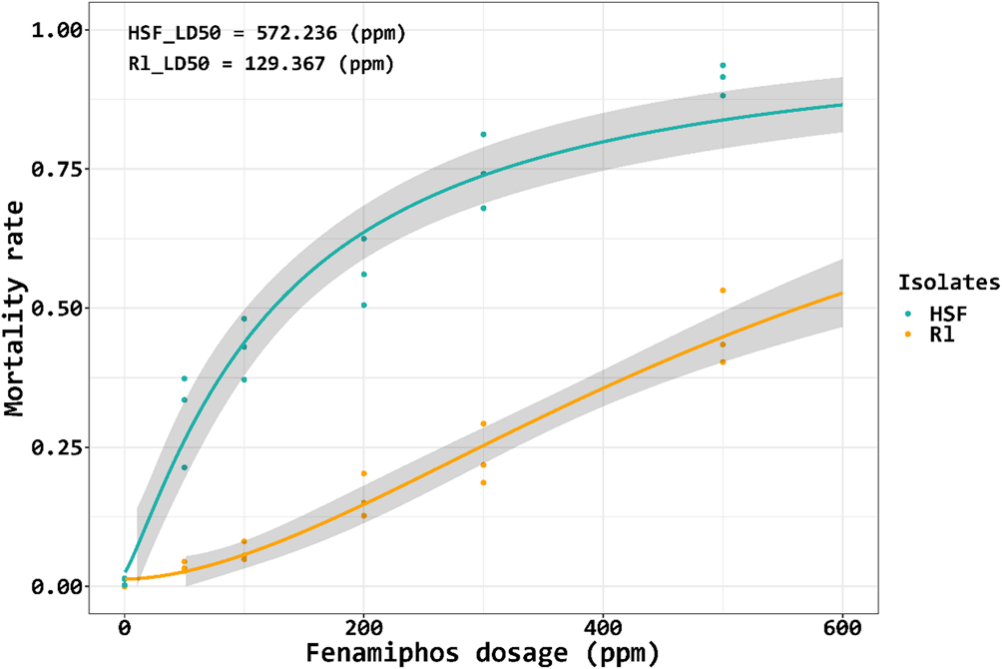
The fenamiphos dose–response curves in correlation to the mortality rates of the *Aphelenchoides besseyi* Rl and HSF isolates. Each point represents the mortality rate of population treated with corresponding fenamiphos dosage in one replicate. Grey bands indicate the degree of confidence. The median lethal doses (LD_50_) of two isolates to fenamiphos are displayed at the top-left corner of the figure.

**Figure 2:**
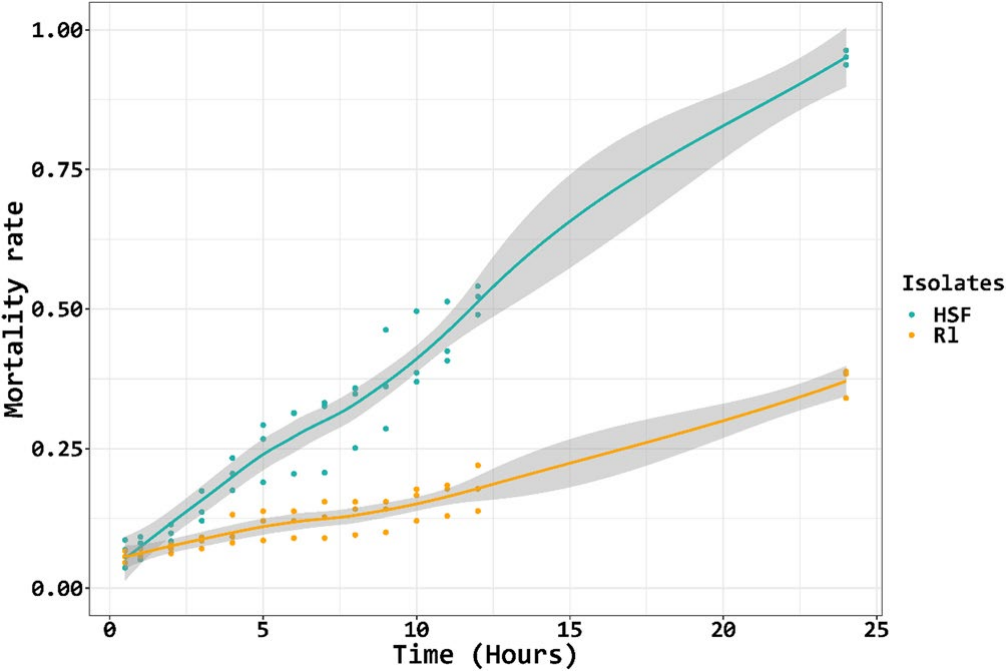
The mortality curves of *A. besseyi* Rl and HSF isolates after being treated with 500 ppm fenamiphos. The mortality rates were recorded at each hour for 12 h and the last data was taken at 24 h post-treatment. Grey bandsindicate the degree of confidence.

Sensitivity assays revealed that the median lethal doses (LD_50_) of Rl and HSF isolates to fenamiphos were 572.2 ppm and 129.4 ppm, respectively. LD_50_ of R1 and HSF to carbofuran were 3702 ppm and 1562.6 ppm, respectively. Fenamiphos dose–response regression curves showed that the Rl isolate was less sensitive to fenamiphos than the HSF isolate (Fig. 1). Similar results were observed when nematodes were treated with carboruran. Analysis of mortality progress curves revealed that the two isolates displayed different responses to 500 ppm fenamiphos in the first 12 h post-treatment (Fig. 2). The curve slope representing the sensitivity of the HSF isolate was much steeper than that of the Rl isolate, indicating further that the Rl isolate was less sensitive to fenamiphos.

### Characterization of three acetylcholinesterase genes in *A. besseyi*

**Figure 3:**
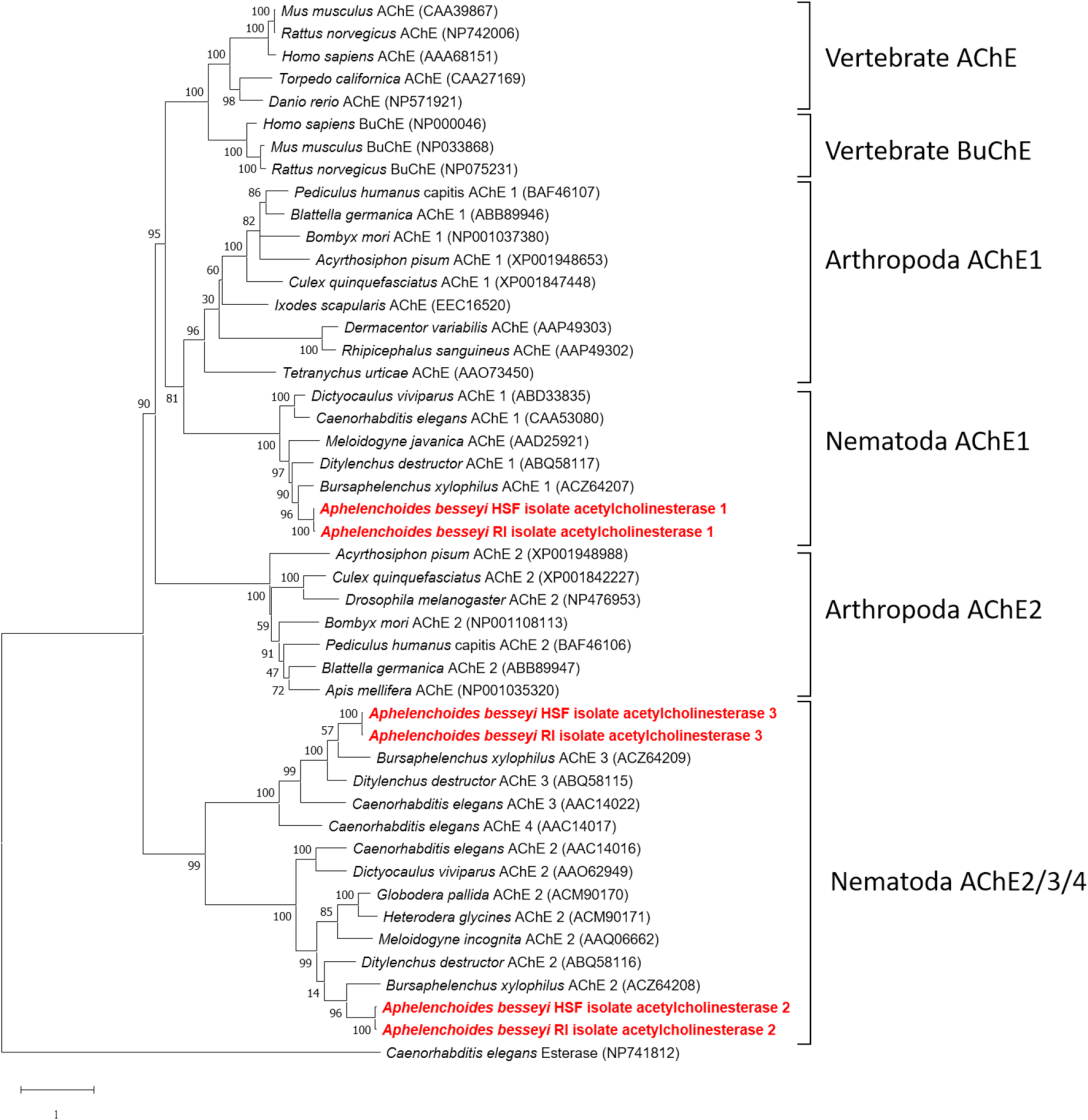
A maximum likelihood phylogeny of six AbeAChEs; *Abace1-Rl* (MT431320), *Abace2-Rl* (MT431322), *Abace3-Rl* (MT431324), *Abace1-HSF* (MT431321), *Abace2-HSF* (MT431323), and *Abace3-HSF* (MT431325) with the AChEs and BuChEs from Vertebrata, Arthropoda and Nematoda. The phylogenetic tree was generated using a ClustalW alignment and maximum likelihood tree with 500 bootstrap replicates using MEGA 7.0.21^47^ (https://mega.software.informer.com/7.0/). GenBank accession numbers of each gene are indicated in parentheses. AbeAChEs are marked in bold and red.

Examination of whole-genome sequences of the R1 isolate identified three *ace* genes designated *Abace1-Rl* (1884 bp, accession no. MT431320), *Abace2-Rl* (1908 bp, MT431322), and *Abace3-Rl* (1815 bp, MT431324) in the genome of the R1 isolate. Three *ace* genes designated *Abace1-HSF* (1887 bp, MT431321), *Abace2-HSF* (1914 bp, MT431323), and *Abace3-HSF* (1815 bp, MT431325) were found in the genome of the HSF isolate. The proteins were named AbAChE1 through AbAChE3. Amino acid sequences of AbAChEs were aligned with 3 BuChEs from vertebrates and 37 AChEs from vertebrates, arthropods and nematodes, and a phylogenetic tree was constructed using esterase of *Caenorhabditis elegans* as an outgroup. The results indicated that each of the *A. besseyi* AChEs was independently grouped with the respective AChEs found in nematodes or other animals (Fig. 3). AbAChE1 of both R1 and HSF isolates was most similar to AChE1 of arthropods, followed by AChE and BuChE of vertebrates.

### Prediction of functional domains of AbeAChEs from two *A. besseyi* isolates

**Figure 4:**
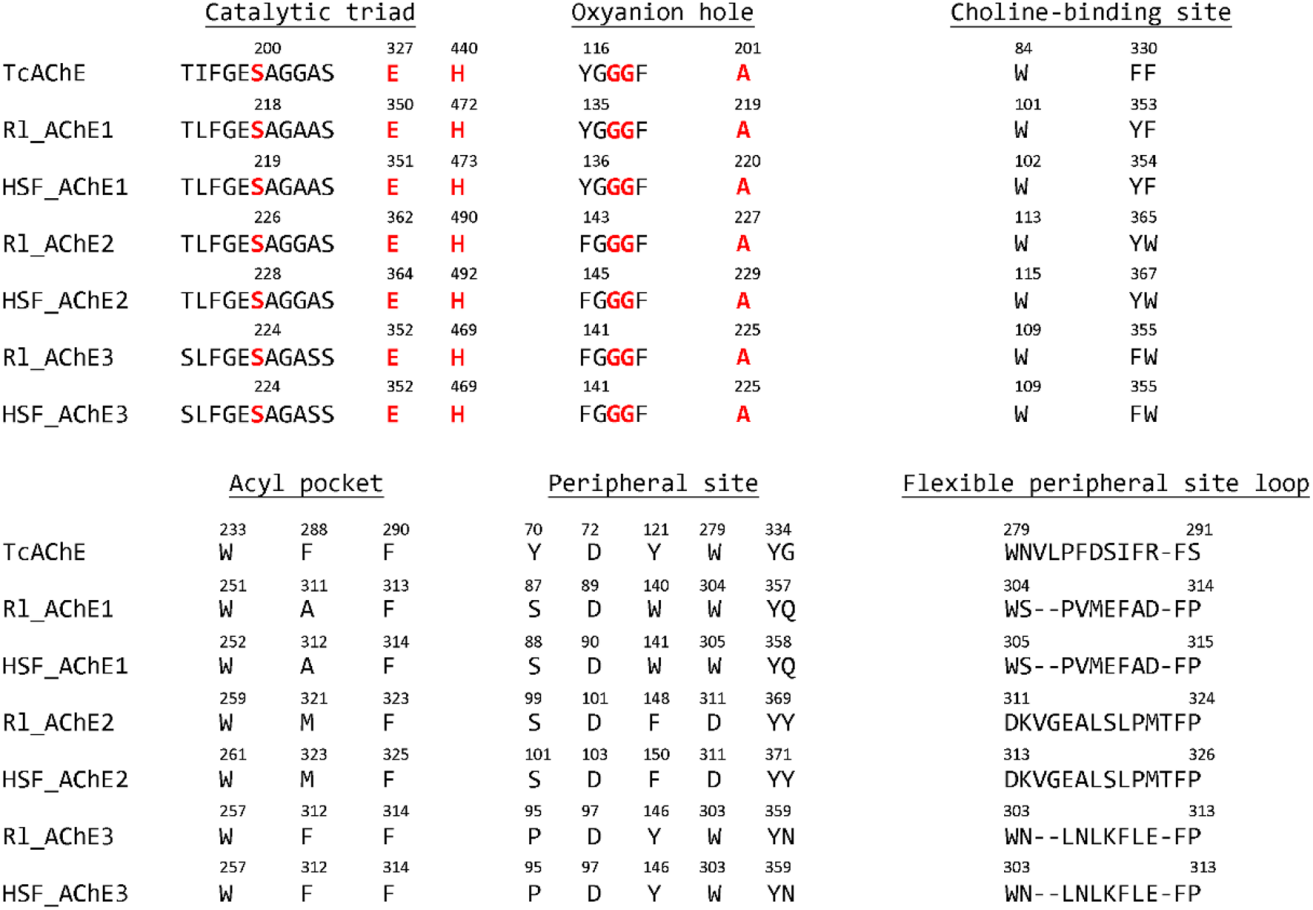
Alignment of AChEs from *Torpedo californica* and *Aphelenchoides besseyi*. Residues marked in red and bold font are essential for catalysis. Numbering of AbeAChEs is based on the protein sequences that translated from the coding sequences.

AbAChEs were found to contain six functional domains: catalytic trial, oxyabion hole, choline-binding site, acyl pocket, peripheral site, and flexible peripheral site loop resembling those found in the *Torpedo californica* AChE (TcAChE) (Fig. 4). Sequence alignment revealed that differences between three AbAChEs were found mainly in the choline-binding site, the Acyl pocket, the peripheral site and the flexible peripheral site loop. No ambiguities were found in the functional domains of AbAChEs between the R1 and the HSF isolates. No substitution was found at catalytic residues in the catalytic triad and oxyanion hole domains between AbeAChEs and TcAChE. Some different amino acid residues were found within choline-binding site and peripheral sites among three AbeAChEs, suggesting different binding abilities. Signal peptides and transmembrane domains were found at the N-termini of AbAChE1 and AbAChE2, but not AbAChE3. Only AbAChE3 was found to have transmembrane domains at the C-terminus, and only AbAChE2 was found to have GPI-anchors at the C-terminus.

### Molecular docking

**Figure 5:**
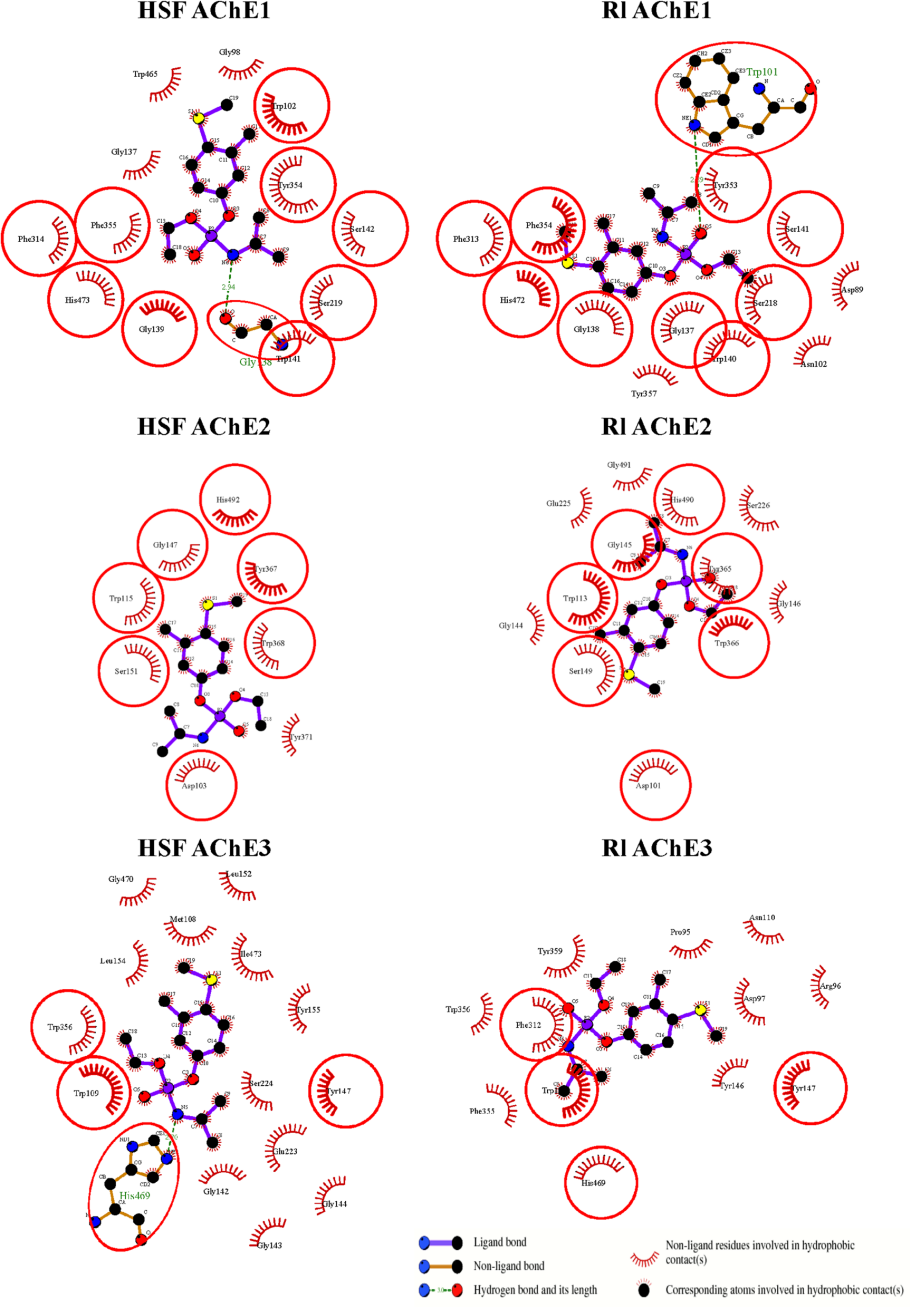
Schematic of possible interactions between fenamiphos and AChE-residues. The key residues surrounding the acetylcholine-binding pocket of AChE1, AChE2, and AChE3 proteins were identified via the best docking poses. The red circles and ellipses indicate the identical residues in both HSF and Rl isolates. The interaction plot was generated by using LigPlot + v.2.2.4 software^60^ (https://www.ebi.ac.uk/thornton-srv/software/LigPlus/).

Molecular modeling was performed to predict 3D structures of three AbAChE proteins and to estimate the binding affinity of fenamiphos in the acetylcholine-binding pocket. Both AbAChE1-HSF ($-$26.79 kcal/mol) and AbAChE3-HSF (AChE3: $-$19.33 kcal/mol) had significantly higher docking scores than AChE1-R1 ($-$2.84 kcal/mol) and AChE3-R1 ($-$5.43 kcal/mol). In contrast, docking scores between AbAChE2-HSF (-22.74 kcal/mol) and AbAChE2-R1 ($-$21.51 kcal/mol) were only slightly different. Further post-docking analyses indicated that several equivalenced residues surrounding the binding pocket in each of AbAChE proteins were predicted to form the hydrogen bonds and hydrophobic interactions (Fig. 5). Fenamiphos could likely engage hydrophobic interactions with Trp102, Tyr354, and Phe355 in AbAChE1-HSF and with Tyr353 and Phe354 in AbAChE1-R1. Those amino acids have been shown to be required for binding to choline according to the model. Fenamiphos might engage hydrophobic interactions with Trp115, Tyr367, and Trp368 in the choline-binding site of AbAChE2-HSF and with Trp113, Tyr365, and Trp366 in AbAChE2-R1. Fenamiphos might engage hydrophobic interactions with Trp109 and Trp356 in the choline-binding site of AbAChE3-HSF and with Trp109, Phe355, and Trp356 in AbAChE3-R1. Fenamiphos was predicted to form hydrogen bonds with Gly138 and Trp101 of AbAChE1-HSF and AbAChE1-Rl, respectively (Fig. 5). In addition, fenamiphos was predicted to form a hydrogen bond with His469 in three AbAChE-HSF proteins, but not in AbAChEs-R1. The results suggested that the binding affinities of three AbAChE-HSF proteins to fenamiphos were higher than those of AbAChE-R1 due to the difference in hydrogen bond force fields, especially in the AbAChE1 and AbAChE3 proteins.

### *Ace* genes were up-regulated after fenamiphos-treating in Rl isolate

**Figure 6:**
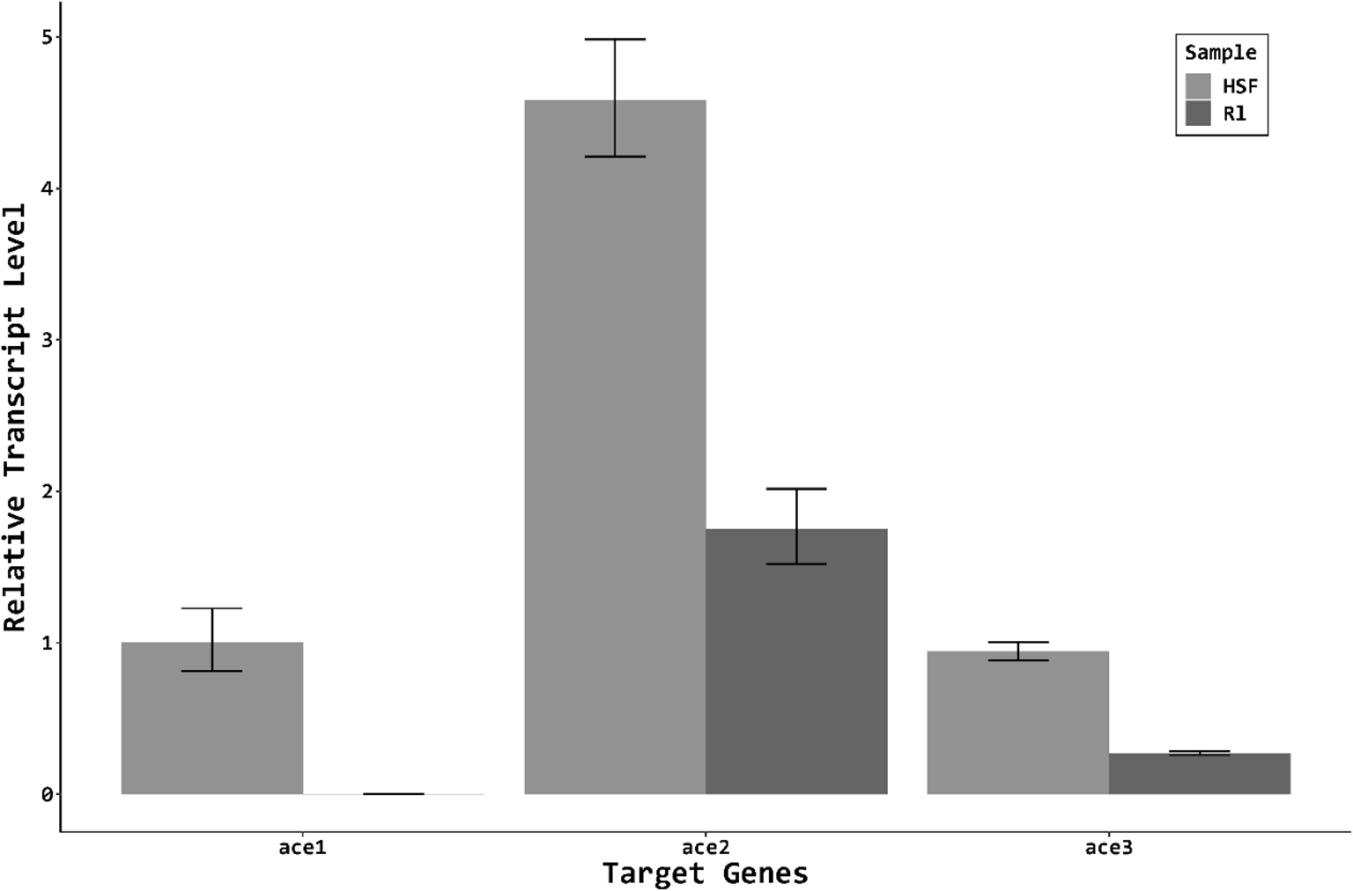
The relative transcript level of the three *ace* genes compared to a reference gene (18 s) in the two *Aphelenchoides besseyi* isolates. The transcript levels were normalized to the HSF AChE1 gene. Error bars represent standard errors. The experiments were performed with three biological replicates, each having three technical replicates.

**Figure 7:**
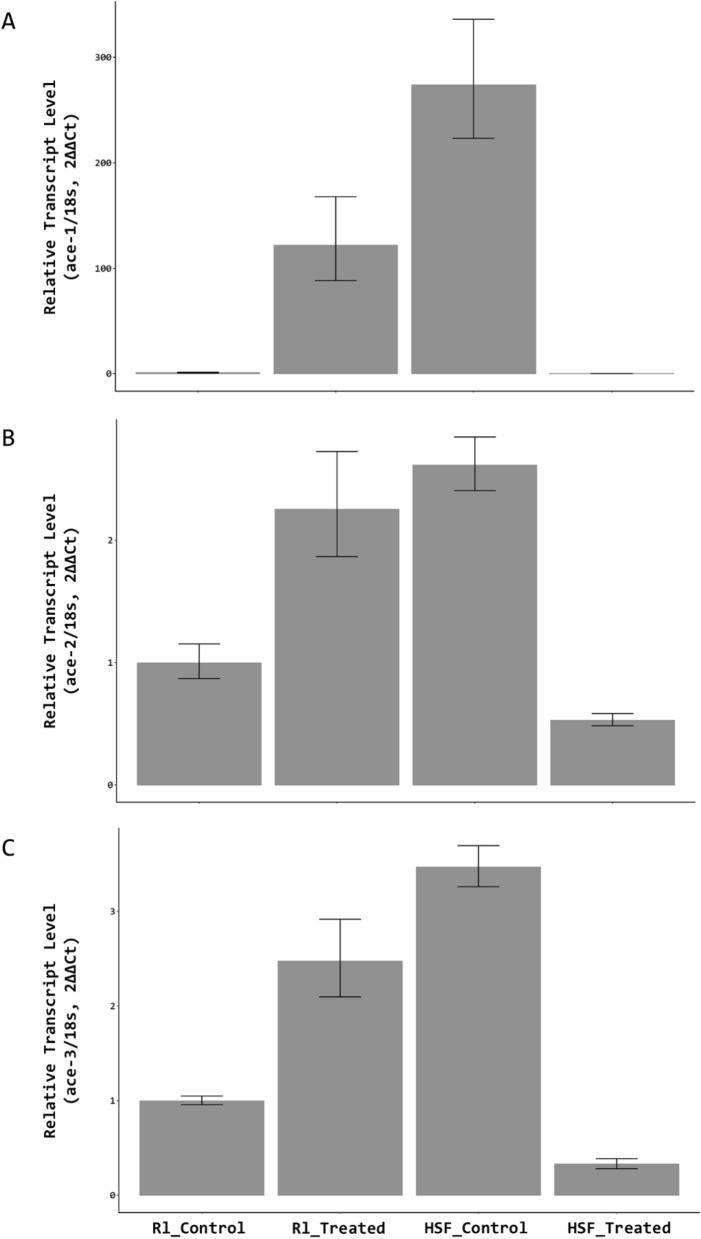
Transcript abundance of three *ace* genes in two *Aphelenchoides besseyi* isolates after being treated with 100 ppm fenamiphos for 12 h. Transcript levels were normalized against the Rl control treatments. Error bars represent standard errors. The experiments were performed with three biological replicates, each having three technical replicates.

The transcript abundance of three *ace* genes were quantified by qRT-PCR. Without fenamiphos treatment, expression of three *ace* genes varied with the highest expression being *Abace2*, followed by *Abace3* and *Abace1* in both Rl and HSF isolates (Fig. 6). Comparing the expression levels between two isolates revealed that expression of the three *Abace* genes was much higher in the HSF isolate than in the R1 isolate, increasing by 274-fold, 2.6-fold and 3.5-fold in terms of *Abace1*, *Abace2*, and *Abace3*, respectively. After fenamiphos treatment, expression of the *Abace* genes was significantly downregulated in the HSF isolate (Fig. 7a-c). In contrast, expression of the *Abace* genes was up-regulated in the Rl isolate after fenamiphos treatment.

## Discussion

### The AChE-inhibitor insensitivity lead to adjustment of nematode disease management

Fenamiphos nematicide is an AChE-inhibiting nematicide commonly recommended for controlling seed-borne rice white-tip nematode *A. besseyi*. In Taiwan, fenamiphos is recommended to be applied by pre-soaking rice seeds in water for 24 h, followed by soaking in 400 ppm fenamiphos for 2 h. However, increasing number of reports indicates that such application often fails to effectively reduce rice white-tip nematode incidences^3,10^. This may be due to the emergence of nematode strains that are resistant to fenamiphos. Our studies have shown that *A. besseyi* obtained from different hosts could have different susceptibilities to the AChE-inhibiting nematicides, further supporting the hypothesis. The Rl isolate originally obtained from a rice plant is highly resistant to fenamiphos and carbofuran (LD_50_ = 572.2 and 3702 ppm, respectively), whereas the HSF isolate originally collected from a fern plant, which has not been exposed to pesticides is less sensitive to the nematicides (LD_50_ = 129.4 ppm and 1562.6 ppm, respectively). Sensitivity tests based on the mortality progress curve have also revealed that the Rl isolate after being treated with 500 ppm of fenamiphos for 2 h only resulted in less than 25% mortality. This indicates that the currently recommended dosage (400 ppm) and treatment duration (2 h) for rice seeds might not be sufficient to kill *A. besseyi*. Further research is warranted to explore appropriate dosage and duration of fenamiphos for effectively controlling *A. besseyi.*

### The AChE genes in *A. besseyi*

In this study, three *Abace* genes in two different *A. besseyi* isolates differing in sensitivity to fenamiphos were sequenced and reported. We have found that *Abeace-1*, *Abeace-2* and *Abeace-3* are orthologous with *ace* genes from other nematodes, and named the gene according to their sequence similarity on the phylogeny clade (Fig. 3). Nematode AChE1 protein is closer to arthropod AChE1, followed by vertebrate AChE and BuChE. Nematode AChE2/3/4 proteins are independently grouped to different clades. In the evolution of AChE, duplication of *ace* loci likely leads to multiple *ace* genes in nematodes during the period of divergence of Protostomia. Nematode AChE2/3/4 have more ancient evolutionary origins than nematode AChE1, which shares the ancestor with arthropod AChE1 and vertebrate AChE^28,32,33^. A recent review on acetylcholinesterase of *Caenorhabditis elegans* has reported that four AChEs perform non-overlapping functions, and each has its own distinct patterns of expression in different organs^34^. The AChE 1/2 localized in neural network and muscle cells, and play non-overlapping roles in synaptic transmission and early development. In contrast, the AChE3 participates in xenobiotic substances defense and other non-neuronal functions, suggesting that functional differentiation may occur in these distinct *ace* loci during evolutionary events. The functions of AChEs can be deduced largely based on the sequence similarity^28,34–36^, which might also apply to the *A. besseyi ace* genes.

### The binding affinities of *A. besseyi* AChEs to AChE-inhibitors

AbeAChEs have all functional domains found in the *Torpedo californica* AChEs (TcAChEs) whose domains, and the co-crystalized structures have been well studied^37,38^. As with TcAChEs, AbeAChEs of both Rl and HSF isolates have conserved catalytic regions. This suggests that AbeAChEs may have similar affinities not only to acetylcholine but also to AChE-inhibitors. Whether or not differences in amino acid residues outside the conserved domains may lead to the disparity of fenamiphos sensitivity between the R1 and HSF isolates remains unknown. The binding affinities of AChEs to fenamiphos deduced from molecular docking analysis suggest that AbAChEs of the Rl isolate have lower affinities to fenamiphos than those of the HSF isolate. This assumption is particularly obvious in the AbAChE1 and AbAChE3 proteins. Lower affinities of AbAChEs to fenamiphos may likely lead to greater resistance as seen in the Rl isolate. In contrast, AbAChEs of the HSF isolate have higher affinities to fenamiphos, which may explain, at least in part, why this isolate is much sensitive to fenamiphos. In addition to conserved domains, AbeAChE1/2 have a signal peptide and a transmembrane domain at their N-termini. AbeAChE3 has a transmembrane domain at C-terminus, and only AbeAChE2 has a GPI-anchor domain at C-terminus. Those domains are not found in AChEs of four reference nematodes: *C. elegans*, *M. incognita*, *B. xylophilus* and *Ditylenchus destructor*.

### Different AChEs basal expression of HSF and Rl isolates

The basal expression of the *Abace* genes is apparently much higher in the HSF isolate than in the R1 isolate (Fig. 6). Studies on human and mice AChEs have revealed that cells displaying high expression levels of AChEs are hypersensitive to AChE-inhibitors ^39^, in those case, the accumulation of AChEs is not significantly induced when exposure to AChE-inhibitors. Further studies are required to determine if the expression levels of *Abace* genes indeed leads to high levels of AChEs and are correlated with the degrees of fenamiphos sensitivity.

### Increasing AChE genes expression levels resulted less sensitivity to AChE-inhibitors

Our results indicated that expression of the *Abace* genes is responding to fenamiphos in a strain-dependent manner in *A. besseyi*. Upon exposure to fenamiphos, all three *Abace* genes in the Rl isolate were significantly upregulated. Studies in greenbug (*Schizaphis graminum*) have found that increasing expression of AChE increases the AChE activity and resistance to organophosphate^24^, and our study also observed similar phenomena. Upregulation of the AChE genes could be via the activation of a transcription regulator. In mice, studies have revealed that the AChE inhibitor activates the c-Fos transcription factor, which in turn regulates the expression of the genes involved in acetylcholine metabolism^40^. Increasing *ace* gene transcripts could also be due to gene duplication, which would lead to up-regulation of the *ace* genes and result in organophosphate resistance^21,23,26^. However, the Rl isolate has only one copy of each of the *ace* genes (unpublished genomic data from Dr. J. I. Tsai). It is unlikely that gene duplication plays a role in the increased expression of *Abace* genes in the R1 isolate in response to fenamiphos.

### Non-neuronal AChE genes involved in chemical defense

Studies in arthropod and nematodes have shown that non-neuronal acetylcholinesterase is responsible for tolerance to pesticides and many xenobiotics^21,29,30^. Knocking down *ace-3* in *B. xylophilus* significantly increases sensitivity to organophosphates and carbamates because the *Bxace-3* product provides the non-neuronal function of chemical defense^29^. Similar studies in *C. elegans* also reveals that up-regulation of *ace-3* results in the detoxification of organophosphate^30^, further confirming the important role of *ace-3* in resistance to organophosphate insecticides. In the present study, we have observed that expression of *ace-3* was upregulated in the R1 strain after fenamiphos treatment, suggesting a possible involvement of detoxification of fenamiphos in the Rl isolate. This could also contribute to the low sensitivity to fenamiphos.

### Downregulated AChE genes resulted in sensitivity to AChE-inhibitor

By contrast, all three *Abace* genes were significantly downregulated in the HSF isolate after fenamiphos treatment. Studies on the expression of the human neuronal acetylcholinesterase coding gene have revealed that dioxin suppresses the expression of the *ace* gene in human neuroblastoma cells via transcriptionally or post-transcriptionally suppression of the aryl hydrocarbon receptor (AhR) pathway or other mechanisms^41,42^. The results further indicate that regulation of the *ace* genes is an intricate process, and we speculate that regulatory mechanisms might be different in the presence or absence of organophosphate^43^.

In summary, discovery of differential sensitivity to fenamiphos nematicide between two closely related isolates of *A. besseyi* has led to explain why fenamiphos fails to effectively control seed-borne rice white-tip nematode in some rice production areas. The two isolates also provide a unique opportunity to investigate possible mechanisms involving in cellular susceptibility or resistance to this nematicide in plant pathogenic nematodes. Our results have suggested that different affinities of AbeAChEs to fenamiphos between two isolates of *A. besseyi*, likely resulting from point mutations, may determine the resistance or susceptibility of the nematode to this nematicide. Different expression patterns of the *Abace* genes in two isolates in response to fenamiphos also suggest that transcriptional regulation of target genes of fenamiphos may contribute to nematode behaviors in response to nematicide.

## Methods

### Collection and identification of nematodes

*Aphelenchoides besseyi* Rl isolate was collected from rice leaves in Linnei Township (Yunlin County, Taiwan). Insecticides and other agricultural chemicals have been regularly applied in that field. The HSF isolate was collected from bird’s nest ferns in Huisun experimental forest (Nantou County, Taiwan), with no history of pesticide application. No approvals were required for the study, which complied with all relevant ethical parameters for plant usage. These two isolates were established by single female according to Jen et al.^44^. Both Rl and HSF isolates were identified to species according to the morphological characters and the 18S rRNA sequences^45^. Nematodes were reared on *Alternaria citri* slant at 27 °C.

### The nematicide bioassays

Fenamiphos, an organophosphate nematicide, and carbofuran (Sigma-Aldrich, St. Louis, USA), a carbamate, was dissolved in acetone, for the following treatments. The nematicide bioassay was modified from the protocol described by Kang et al.^7^ Fenamiphos was diluted to five concentrations: 5000, 10,000, 20,000, 30,000 and 50,000 ppm, and carbofuran 500, 5000, 10,000, 15,000 ppm. Nematodes were washed out from a slant using 2-ml ddH_2_O. Nematode suspensions (495 μl) containing approximately 150 nematodes were mixed with 5 μl fenamiphos or carbofuran in a 1.5 ml microcentrifuge tube. The final concentrations of fenamiphos were 50, 100, 200, 300, 500 ppm, and carbofuran 50, 500, 1000 and 1500 ppm. The nematodes treated with the nematicides were incubated at 27℃ for 24 h on a shaker in the dark, and the mortality rates were recorded. Nematodes that were rigidity and remained motionless for 3 s after touching were presumed dead. Each treatment had three replicates, and the experiment was repeated three times. The data was plotted as dose–response curves.

The mortality progress curve of both R1 and HSF isolates was obtained as follows. The mortality rates of nematodes after being treated with 500 ppm of fenamiphos were recorded every hour for 12 h, and the last data was taken at 24 h post-treatment. Each isolate had three replicates and the experiment was repeated three times.

The dose–response regression curves with standard error and the median lethal doses (LD_50_) were deduced by the ‘drc’ package^46^ in R environment. Dose–response regression curves and mortality progress curves were plotted by ‘ggplot2’ package.

### Generation of cDNA

All stages of *A. besseyi* were washed with sterile distilled water from slant culture medium and purified by the modified Bearmann funnel technique for 12 h before total RNA extraction. Nematodes were mixed with 100 ppm fenamiphos and incubated at 27℃ for 12 h on a shaker in the dark. Nematodes were washed two times with ddH_2_O before RNA extraction using GENEzol™ TriRNA Pure Kit (Geneaid, New Taipei City, Taiwan) according to manufacturer’s instructions. First stranded cDNA was synthesized using SuperScript III Reverse Transcriptase (Invitrogen, California, USA) using an oligo-dT primer.

### Identification of acetylcholinesterase (*ace*) genes in *A. besseyi*

The *Bursaphelenchus xylophilus ace* genes (Accession Nos: ACZ64207.1, ACZ64208.1, ACZ64209.1) and the *Ditylenchus destructor ace* genes (Accession Nos: ABQ58117.1, ABQ58116.1, ABQ58115.1) were used as queries to search against *A. besseyi* transcriptome database (Accession: SRX385206) to identify the reserved regions of the *ace* genes. The primers (Table S1) used for the following experiments were designed based on the conserved regions. The 3’ and 5’ cDNA ends flanking the acetylcholinesterase coding gene were obtained by 3’ RACE and 5’ RACE for the Rapid Amplification of cDNA Ends (Invitrogen, Carlsbad, California, USA, #18373-019 and #18374-058). The resultant PCR fragment was used as a template for amplification with a set of nest primers in the same tube. Amplification started with denaturation at 94 °C for 3 min, followed by 35 cycles of 94 °C for 45 s, 58 °C for 45 s, and 72 °C for 1 min 30 s, and a final extension at 72 °C for 10 min. PCR products were separated in 1.2% agarose gels and stained with 40 ppm ethidium bromide solution. DNA fragments were recovered from gel with the Gel Elution Kit (GeneMark, Taipei, Taiwan, #DP03) and cloned into a vector with the TOPO TA Cloning kit (Invitrogen, Carlsbad, California, USA). Plasmids were propagated in *Escherichia coli* DH5α cells, extracted, and sequenced at NCHU Biotechnology Center (Taichung, Taiwan). Each of the *ace* genes with three biological repeats from two *A. besseyi* isolates were sequenced, and three independent clones of each gene were sequenced in each repeat.

### Bioinformatics analysis

Open Reading Frame Finder (ORF Finder, https://www.ncbi.nlm.nih.gov/orffinder/) was used to identify the start and stop codons of *ace* genes encoding Acetylcholinesterase (AChE). Deduced protein sequences were used for phylogenic analysis and prediction of functional domains. AChEs from other vertebrates, nematodes and arthropods used in the phylogenic analysis^35^ were downloaded from NCBI database. Multiple alignment analysis was performed by ClustalW with default parameters. The phylogenetic tree was constructed using the Maximum Likelihood method with LG model and Gamma Distributed (G) with 500 bootstrap replicates^47–49^ using MEGA 7.0.21. (https://mega.software.informer.com/7.0/)47 AChEs were aligned with AChEs from *Torpedo californica*, *Homo sapiens*, and *Drosophila melanogaster* (SwissProt codes: ACES_TETCF, ACES_HUMAN and ACES_DROME) to identify functional domains previously reported in AChE^50^. Transmembrane domains, signal peptides and Glycosylphosphatidylinositol (GPI) Anchors were predicted by TMHMM Server v. 2.0, SignalP-5.0 Server and PredGPI GPI-Anchor Predictor, respectively^51–54^.

### Molecular docking evaluation

The 3D structures of three AChE proteins obtained from *A. besseyi* HSF and Rl isolates were homology-modeled using the I-TASSER standalone package version 5.1^55^ (https://zhanggroup.org/I-TASSER/download/) with default parameters. The simulated structures were applied in the molecular docking study using Discovery Studio 2016 software (BIOVIA, San Diego, CA, USA)^56^ to assess possible binding modes of fenamiphos in AChE active site. The initial structures were prepared using the Prepare Protein protocol in Discovery Studio to insert missing loop regions based on SEQRES data and to protonate the structures at pH 7.4. The crystal structure of *Torpedo californica* AChE in complex with acetylcholine (PDB code: 2ACE)^57^ was employed as a reference structure to determine the docking site through the superimposition of the simulated structures onto the co-crystallized structure. The docking was performed using the CDOCKER^58^ docking protocol in Discovery Studio and potentials were added applying the CHARMM force field^59^. Conformations of fenamiphos were generated via random rotations and high-temperature molecular dynamics and were refined by grid-based simulated annealing and minimization. The best docking poses were selected following the highest docking energy scores. The post-docking analyses were performed using LigPlot + version 2.2.4 software^60^ (https://www.ebi.ac.uk/thornton-srv/software/LigPlus/) to identify the ligand–protein hydrogen bonds and hydrophobic moieties.

### Quantitative RT-PCR (qRT-PCR)

qRT-PCR was performed to assess the expression levels of the *ace* genes in two different *A. besseyi* isolates treated with or without 100 ppm fenamiphos using CFX Connect Real-Time PCR Detection System (Bio-Rad Laboratories, Inc). Expression of the 18 small-subunit ribosomal (18S) gene was used as the internal reference gene in the experiment. Oligonucleotide primers used for each gene are listed in Table S1. For each primer pair, PCR efficiencies were determined by standard curves. The reactions started at 95℃ for 3 min, followed by 40 cycles of 95℃ for 10 s and 59.5℃ for 30 s. Efficiencies of primer pairs for genes in Rl isolate were 83.5% (18S), 96.4% (*ace1*), 96.6% (*ace2*), and 99.3% (*ace3*). In HSF isolate, efficiencies of primer pairs were 83.7% (18S), 92.2% (*ace1*), 95.5% (*ace2*), and 90.3% (*ace3*) (all with R^2^ ≥ 0.999). Concentrations of each cDNA were adjusted to about 1 ng/μl. Four genes (18S, *ace1*, *ace2* and *ace3*) from two nematode isolates with three technical replicates and three biological replicates were analyzed by qRT-PCR. Data were analyzed with the ΔΔCT method^61,62^ and compared between mock and fenamiphos-treated groups by one-tailed Student’s *t*-test. Data after analyzing by the ΔΔCT method were converted to fold changes, and plotted in R environment by ‘ggplot2’ package.

## Supplementary Information

Supplementary Information.
