# Supplementary material for: The ACE genes in Aphelenchoides besseyi isolates and their expression correlation to the fenamiphos treatment
Source: Sci Rep. 2022 Feb 7;12:1975. doi: 10.1038/s41598-022-05998-y (PMC8821594; doi:10.1038/s41598-022-05998-y)
Supplement: Supplementary file 1 — Supplementary Information. [file 41598_2022_5998_MOESM1_ESM.docx]

Supplement Table S1 Primers used for *Aphelenchoides besseyi* cDNA, genomic DNA and qRT-PCR analysis

| primer names | Target gene | Sequence (5'-3') | Description |
| --- | --- | --- | --- |
| HSF-ace1-RACE | Ace-1 | GCCGGTTCAAATTTGGAGGA | 3' RACE |
| HSF-ace1-NEST | Ace-1 | GTTGGCTGATGTGTTTCCACC |  |
| Rl-ace1-RACE | Ace-1 | AGGTTTTCCACCGGAACCTC |  |
| Rl-ace1-NEST | Ace-1 | CACGGACCGAGACGAAAAGA |  |
| Ace2-RACE | Ace-2 | CAGCATTATGATGGAGTGCG |  |
| Ace2-NEST | Ace-2 | GAATCCACCGAACGAGTTGGAT |  |
| Rl-ace3-RACE | Ace-3 | TGTGGCTTTGCACGAAAAGAA |  |
| HSF-ace3-RACE | Ace-3 | GGTTGGCAGATCAAATGAGTG |  |
| Ace3-NEST | Ace-3 | CGGAACAACCGCTTTTGACA |  |
| AUAP |  | GGCCACGCGTCGACTAGTAC |  |
| adapter primer |  | GGCCACGCGTCGACTAGTACTTTTTTTTTTTTTTTTT |  |
| HSF-ace1-RACE | Ace-1 | ATCGCCTGTAACGCTAACGAC | 5' RACE |
| HSF-ace1-NEST | Ace-1 | ACTTCTCAGCCAGACATCACG |  |
| Rl-ace1-RACE | Ace-1 | ACGGATAAACGACTCGCCAA |  |
| Rl-ace1-NEST | Ace-1 | CAATTCGCTGAGCACCGTTT |  |
| Ace2-RACE | Ace-2 | ATCCAACTCGTTCGGTGGATTC |  |
| Ace2-NEST | Ace-2 | GTGGAAACGCGGTGTCAATC |  |
| Ace3-RACE | Ace-3 | CTGCTCGGTTGGTCGAAGTA |  |
| Ace3-NEST | Ace-3 | CAAAGGCGGTCTGAACACAC |  |
| Poly G |  | GGGGGGGGGGGAAAGGG |  |
| Poly C |  | CCCTTTCCCCCCCCCCC |  |
| Ace1-FL-F | Ace-1 | AAGAGGCACGGGATGAACTG | cDNA amplification |
| Ace1-FL-R | Ace-1 | TAGCACACTGACCCTGTTGC |  |
| HSF-Ace2-FL-F | Ace-2 | ACGGACGATGTCGTTGTGAA |  |
| Rl-Ace2-FL-F | Ace-2 | GGGGTCCAGTTGAATTTCCGAAGAT |  |
| Ace2-FL-R | Ace-2 | GGTTTATTTTGTACATGAAGGCGGTAGT |  |
| Ace3-FL-F | Ace-3 | GCTTTTCGACCATCGACTTGG |  |
| Ace3-FL-R | Ace-3 | TTGGTTGTTTCGCATTCACCG |  |
| 18S-qPCR-F | 18s ribosomal DNA | TAGAGCGAAACTGCGAACGG | qRT-PCR |
| 18S-qPCR-R | 18s ribosomal DNA | AATAGCACTGCGACCCGAAG |  |
| Rl-ace1-qPCR-F | Ace-1 | CACATCCACAGCTCACGGTAAA |  |
| Rl-ace1-qPCR-R | Ace-1 | CTCCAAAGTACGTGTCCAGAGATTG |  |
| Rl-ace2-qPCR-F | Ace-2 | GTGCGATTCGTGGACTGGAAC |  |
| Rl-ace2-qPCR-R | Ace-2 | GCCAATAGTTCACCTTCCCAACG |  |
| Rl-ace3-qPCR-F | Ace-3 | CTTCGATTCCGTCCACCGATTG |  |
| Rl-ace3-qPCR-R | Ace-3 | TCGGCTGGTGACCAAATGTTC |  |
| HSF-ace1-qPCR-F | Ace-1 | GAATGGTCGCCAGTGATGGA |  |
| HSF-ace1-qPCR-R | Ace-1 | TGAACCGGCAAGTAGTTGGG |  |
| HSF-ace2-qPCR-F | Ace-2 | GGCGAAGCTCTTTCATTGCC |  |
| HSF-ace2-qPCR-R | Ace-2 | CATTGTGCCAGCAAGTACCG |  |
| HSF-ace3-qPCR-F | Ace-3 | ACCGCTGGATACACCAATCG |  |
| HSF-ace3-qPCR-R | Ace-3 | TTCAAATGCATCCAACGGCG |  |
